# Do we truly understand pollination syndromes in *Petunia* as much as we suppose?

**DOI:** 10.1093/aobpla/ply057

**Published:** 2018-10-01

**Authors:** Daniele M Rodrigues, Lina Caballero-Villalobos, Caroline Turchetto, Rosangela Assis Jacques, Cris Kuhlemeier, Loreta B Freitas

**Affiliations:** 1 Laboratory of Molecular Evolution, Department of Genetics, Universidade Federal do Rio Grande do Sul, Porto Alegre, Rio Grande do Sul, Brazil; 2 Department of Inorganic Chemistry, Universidade Federal do Rio Grande do Sul, Porto Alegre, Rio Grande do Sul, Brazil; 3 Institute of Plant Sciences, Altenbergrain, Bern, Switzerland

**Keywords:** Anthocyanins, chemical ecology, flavonols, pollinator attraction, *Pseudagapostemon*, scent, UV-light response

## Abstract

*Petunia* is endemic to South America grasslands; member of this genus exhibit variation in flower colour and shape, attracting bees, hawkmoths or hummingbirds. This group of plants is thus an excellent model system for evolutionary studies of diversification associated with pollinator shifts. Our aims were to identify the legitimate pollinator of *Petunia secreta*, a rare and endemic species, and to assess the importance of floral traits in pollinator attraction in this *Petunia* species. To determine the legitimate pollinator, field observations were conducted, and all floral visitors were recorded and evaluated. We also measured the nectar volume and sugar concentration. To characterize morphological cues for pollinators, we assessed the ultraviolet (UV)-light response in detached flowers, and characterized the floral pigments and pollen volatile scents for four different *Petunia* species that present different pollination syndromes. *Petunia secreta* shares the most recent ancestor with a white hawkmoth-pollinated species, *P. axillaris*, but presents flavonols and anthocyanin pigments responsible for the pink corolla colour and UV-light responses that are common to bee-pollinated *Petunia* species. Our study showed that a solitary bee in the genus *Pseudagapostemon* was the most frequent pollinator of *P. secreta*, and these bees collect only pollen as a reward. Despite being mainly bee-pollinated, different functional groups of pollinators visit *P. secreta*. Nectar volume, sugar concentration per flower, morphology and components of pollen scent would appear to be attractive to several different pollinator groups. Notably, the corolla includes a narrow tube with nectar at its base that cannot be reached by *Pseudagapostemon*, and flowers of *P. secreta* appear to follow an evolutionary transition, with traits attractive to several functional groups of pollinators. Additionally, the present study shows that differences in the volatiles of pollen scent are relevant for plant mutualistic and antagonist interactions in *Petunia* species and that pollen scent profile plays a key role in characterizing pollination syndromes.

## Teaser

Petunia secreta is a species that can be found in just few sites in the south of South America. This species displays several morphological traits that are associated with attracting bees, such as pink coloured petals, but also presents characteristics that are attractive to other pollinators. In this study we identified the legitimate pollinator of P. secreta and used several different approaches to demonstrate that the interaction between Petunia species and their pollinators is more complex than has been thought.

## Introduction

Pollination syndromes were first defined by Federico Delpino (Fenster *et al.* 2004) as suites of floral traits associated with particular pollinator groups (Etcheverry and Alemán 2005). Animal pollinators have acted as drivers of floral diversification and plant speciation (van der Niet and Johnson 2012; van der Niet *et al.* 2014), and the pollinators that most frequently visit and efficiently pollinate the flowers select for a number of floral traits in the long term (Armbruster 2014). In this sense, it is expected that similar suites of floral traits can reflect convergent adaptation to a particular type of pollinator in distantly related taxa (Proctor *et al.* 1996; Fenster *et al.* 2004).

Pollinators are agents of directional selection on interlinked floral traits such as colour and volatiles (Yan *et al.* 2016). Greater effectiveness in insect landing is promoted when visual cues and olfactory signals from the pollen are combined (Lunau 1992). Floral volatiles play an important role in attracting pollinators (Raguso 2001; Knudsen and Gershenzon 2006; Knudsen *et al.* 2006); scent stimuli are learned more quickly than visual clues in bees (Arenas and Farina 2014) and may differentially attract certain pollinator species (Huber *et al.* 2005; Klahre *et al.* 2011).

*Petunia* is a young lineage in the Solanaceae family; it comprises species from subtropical and temperate South America. These species display flowers with different colours and shapes (Stehmann *et al.* 2009) and have bees, hawkmoths or hummingbirds as pollinators (Gübitz *et al.* 2009; Knapp 2010). The genetic architecture of floral syndromes has been studied in the *Petunia*, revealing genes associated with floral traits, especially volatile emissions and ultraviolet (UV)-light reflectance (Amrad *et al.* 2016; Sheehan *et al.* 2016). These features make *Petunia* a suitable model system for investigating pollinator-driven divergence and a good example of how key floral traits that affect pollinator behaviour can lead to reproductive isolation and adaptation (Gübitz *et al.* 2009; Fregonezi *et al.* 2013; Vandenbussche *et al.* 2016).

When plant species that do not present intrinsic (post-pollination) reproductive barriers occur sympatrically, they usually exhibit different floral signals attracting different pollinator species (Huber *et al.* 2005). Previous studies have suggested that selection for different pollinators is an important force driving floral diversification in *Petunia* (Fregonezi *et al.* 2013). In addition, the most recent molecular phylogeny of the genus (Reck-Kortmann *et al.* 2014) supports two main clades mainly related to differences in the corolla tube length. The first clade includes 11 bee-pollinated species presenting short corolla tubes, pink flowers and blue pollen. The second clade includes three species with long corolla tubes and yellow pollen (*Petunia axillaris*, *P. exserta* and *P. secreta*) with remarkably diverse pollination syndromes and corolla colours. Basal to this long-tube clade arises *Petunia occidentalis*, which displays traits of typical species included in the first clade.

The long-tube *Petunia* species exhibit diverse flower morphologies and pollinators. Plants of *P. axillaris* have white flowers, produce floral scents at night and are moth-pollinated (Galliot *et al.* 2006; Venail *et al.* 2010); *P. exserta* has red flowers, with anthers and stigmas conspicuously exerted from the corolla, and pollination by hummingbirds (Lorenz-Lemke *et al.* 2006; Stehmann *et al.* 2009). *Petunia secreta* has pink flowers, and bees have been suggested as the probable pollinators, based on the flower morphology and some informal observations (Stehmann and Semir 2005).

Several studies made under garden conditions have identified the floral traits that attract pollinators in a few *Petunia* species. Using molecular tools and comparisons of pollinator behaviour and preferences, these studies showed that in *P. axillaris*, *P. inflata*, *P. integrifolia* and *P. exserta* the flower morphology, scent emission, nectar composition and UV-light reflectance are involved in the specialization to different pollinators and consequently in species diversification (Hoballah *et al.* 2007; Venail *et al.* 2010; Hermann and Kuhlemeier 2011; Klahre *et al.* 2011; Sheehan *et al.* 2012, 2016; Dell’Olivo and Kuhlemeier 2013; Gleiser *et al.* 2014; Hermann *et al.* 2015).

Despite these previous studies, the literature on reproductive biology, pollinator attraction and evolutionary aspects of plant animal interactions remains scarce for the majority of *Petunia* species in natural conditions. For example, although floral scent appears to play an important role in reproductive isolation in *Petunia* (Verdonk *et al.* 2005; Hoballah *et al.* 2007; Klahre *et al.* 2011; Kessler *et al.* 2013; Amrad *et al.* 2016), information on natural populations is scarce regarding scent chemistry across the genus. Data on the pollen scent composition of *Petunia* are still unavailable, and could further elucidate the plant–pollinator interactions for *Petunia* species.

*Petunia secreta* is an interesting species because it belongs to the clade that presents the greatest floral variation in the genus, probably driven by pollinators (Fregonezi *et al.* 2013). This species, with its pink and non-fragrant corolla (Stehmann and Semir 2005), diverged recently from the large and white-flowered *P. axillaris* (Reck-Kortmann *et al.* 2014). Though not being found in exactly the same sites, *P. secreta* and *P. axillaris* generally occur in the same geographical region (Turchetto *et al.* 2015a; Rodrigues *et al.* 2018).

In this study, we evaluated the floral biology of *P. secreta* by recording its floral traits (nectar, petal colour and pollen scent); we also made field observations for flower visitors and legitimate pollinators, as well as recorded their foraging behaviours. In addition, we compared the pollen scent and floral features among species showing variation associated with pollination syndromes: *P*. *secreta* (possibly bee-adapted), *P*. *axillaris* (moth-adapted), *P*. *exserta* (hummingbird-adapted) and *P*. *integrifolia* (bee-adapted). We wished to establish whether there is any relationship between these traits and the known or predicted pollinators for these species.

We have two main aims in this study: (i) a detailed study of pollination of one species, *P. secreta*, including description of pollinator-attraction traits, and (ii) a comparative study of pollen scents in four *Petunia* species with different pollination syndromes. Our questions were as follows: (i) What is the legitimate pollinator of *P. secreta*? (ii) Does *P. secreta* offer rewards to its pollinators? (iii) How can its floral attributes affect pollinator attraction? (iv) What is the chemical composition of the pollen volatiles in different *Petunia* species that present different pollen colour and different pollinators? (v) Can pollen fragrance profiles and nectar provide information for predicting pollinators in *P. secreta* and be useful to understand diversification in the genus?

## Methods

### Studied species and area

*Petunia secreta* is annual and blooms from September to December (spring in the South Hemisphere) similarly to other *Petunia* species. The *P. secreta* corolla consists of a long tube that flares into a trumpet. It is pink, and the anthers are yellow (Stehmann and Semir 2005). This species is endemic to a low-elevation mountain range in a region known as Serra do Sudeste (Stehmann *et al.* 2009) and is an endangered species according to IUCN criteria. Two lineages associated with different environments were found by Turchetto *et al.* (2016). In the Serra do Sudeste, *P. secreta* co-occurs with *P. axillaris*, *P. exserta* and *P. integrifolia* although each species inhabits different sites.

In the greenhouse, *P. secreta* flowers remain open for 4 days if not pollinated, and flower senescence is characterized by changes in the colour of the corolla, followed by the gradual wilting of the petals. Anther dehiscence occurs simultaneously with the opening of the flower (within ~30 min), and the anthers are always positioned below the stigma. *Petunia secreta* is self-compatible, but it cannot spontaneously self-pollinate (Rodrigues *et al.* 2018).

**Figure 1.**
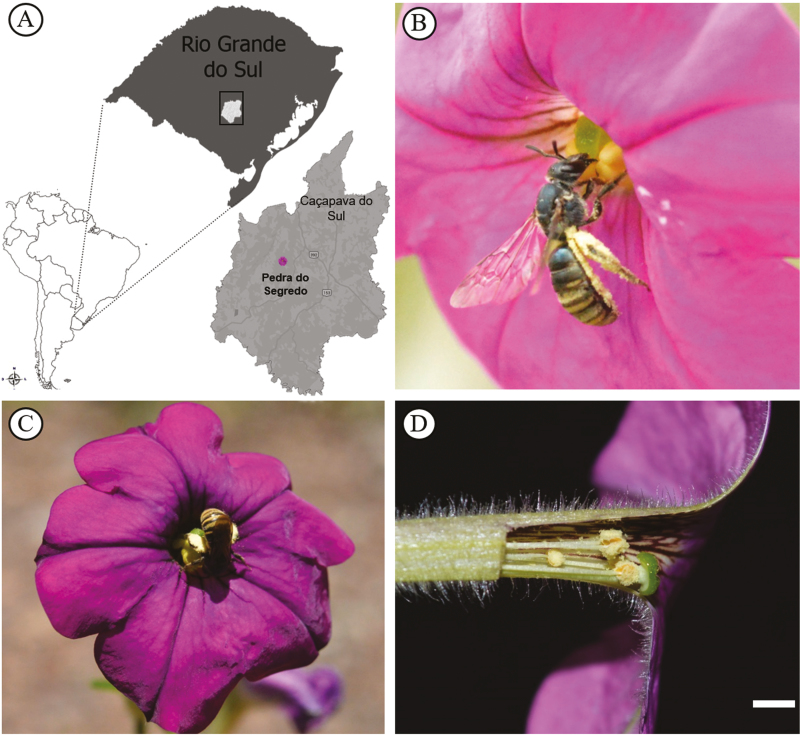
*Petunia secreta*: A, collection site; B, legitimate pollinator (*Pseudagapostemon* sp. bee) on flower; C, *Pseudagapostemon* sp. positioning for effective pollination in *P. secreta*; D, reproductive organs of *P. secreta* highlighting the anthers and stigma position and differences in anther length. Bar = 1 cm.

We carried out the experiments in Caçapava do Sul municipality in the central region of the Rio Grande do Sul Brazilian state (Fig. 1A), ca. 350 m in elevation. During the spring of 2 years (September to December 2014 and 2015), we visited the region to observe pollinators. To minimize the impact on the natural populations, seeds were collected and germinated in growth chambers to obtain plants that were then cultivated in a greenhouse following the protocols of Rodrigues *et al.* (2018). Various traits were investigated in the cultivated plants, such as nectar (volume and sugar concentration), stigmatic receptivity, flower UV-light response and floral pigments **[see****Supporting Information—Table S1****]**.

### Nectar traits and stigmatic receptivity

We measured the volume and sugar concentration of nectar from five flowers from each of four individuals. The flower buds were bagged, and nectar volumes were extracted 24 h after opening of the flower with a graded 25 µL volume Hamilton microsyringe (Sigma-Aldrich Co., St. Louis, MO, USA). The sugar concentration was measured with a portable refractometer. Stigma receptivity was tested in five individuals by plunging the stigmatic surface into 100 % hydrogen peroxide P.A. (Merck & Co., Kenilworth, NJ, USA) at 100 %. The tests were performed in four stages using 10 flowers per stage: pre-anthesis floral buds; flowers after anthesis immediately after the opening of the anthers; flowers in which the corolla colour was starting to change (pink to purple, indicating the early flower senescence stage); and flowers with wilted petals. A positive result was observed when oxygen bubbles resulting from stigma-hydrogen peroxide reactions were produced (according to Zeisler 1938).

### UV-light response

For the UV-light response experiments, we used flowers from greenhouse-grown plants of *P. secreta*, *P. axillaris*, *P. exserta* and *P. inflata*. These species represent different floral morphologies and all pollination syndromes described in *Petunia*. We obtained images of detached flowers with UV light using a Nikon 60 mm 2.8D microlens and a Nikon D7000 SLR camera (Nikon Co., Tokyo, Japan) that was converted to record UV light by replacing the manufacturer’s filter with a UV-specific filter that blocked visible and infrared light (Advanced Camera Services Ltd, Watton, UK). A Metz MZ76 flashgun (Metz-Werke GmbH & Co. KG, St. Chandler, AZ, USA) that was modified to produce UV-A light (320–390 nm; Advanced Camera Services Ltd) provided the light source. Images were converted to greyscale in Photoshop CS5 (Adobe Systems Co., San Jose, CA, USA) and, when necessary, exposure was adjusted over the complete image. Flowers were scored either as UV-absorbent or UV-reflective based on comparison with the UV-absorbent positive control, a *P. axillaris* flower (Sheehan *et al.* 2016).

### Spectrophotometric quantification of flavonols and anthocyanins

We used petals of cultivated individuals of *P. secreta*, *P. axillaris*, *P. exserta* and *P. integrifolia* growing under the same conditions to quantify the flavonols and anthocyanin floral pigments. For each species, we sampled discs from the corolla limb (8 mm in diameter) of one flower from each of three different individuals, put each disc into 1 mL of extraction buffer (2:1:7 methanol:acetic acid:water) and kept the solution in the dark for 48 h (modified from Ando *et al.* 1999). A spectrophotometer SpectraMax M4 (Carl Zeiss AG, Oberkochen, Germany) was used to measure the absorption spectra. Flavonols levels are detected at 300–385 nm (Tsimogiannis *et al.* 2007), whereas anthocyanins are detected at 400–600 nm (Merzlyak *et al.* 2008).

### Pollen scent composition

Plants used for scent collection were selected at random from populations in the field. We collected anthers of *P. secreta*, *P. axillaris*, *P. exserta* and *P. integrifolia*. For each species, anthers of 10 flowers of different individuals were gathered in the same flowering season (November 2015) in sealed tubes 1 h after anthesis (~11:00 AM).

Volatile compounds from the pollen were determined out using the headspace solid-phase microextraction method (HS-SPME; Supelco Inc., Sigma-Aldrich) and gas chromatography-mass spectrometry (GC/MSD). The peak area of each compound was used for quantification. A 100-µm polydimethylsiloxane divinylbenzene (PDMS/DVB) fibre was used. After 5 min of sample conditioning, the SPME fibre was exposed in the headspace for 30 min, and then immediately inserted into the GC–MS injector port at 250 °C for 5 min. The MSD data were used for compound identification based on comparison of the mass spectra with those from the National Institute of Standards and Technology (https://www.nist.gov) and their retention indexes with the published data.

Volatile components were first classified into different chemical categories: phenylpropanoids, benzenoids, mono- and sesquiterpenes, nitrogen-containing compounds and aliphatic alcohols (Knudsen *et al.* 1993, 2006), which allowed better visualization of the variation in molecular compounds. Then, we performed a similarity analysis of the volatile organic compounds (VOCs) based both on a presence/absence matrix and on the quantitative measures. We conducted a principal component analysis (PCA) using the *prcom* function of the package stats in R v.3.5.0.

### Observation of floral visitors

We observed floral visitors to flowers of *P. secreta* in the field at Pedra do Segredo (Fig. 1A). We selected this site because the population comprises several individuals with several flowers per individual each season, which is not always the case in *P. secreta* (Turchetto *et al.* 2016; Rodrigues *et al.* 2018). Initially, we patrolled the population and gathered information about all possible visitors from 08:00 AM to 07:00 PM for 2 days. As visitors were not observed after 6:00 PM and *P. secreta* reflects UV light (see Results), we restricted the observations for this study to daytime only. Subsequently, diurnal visitors were recorded, and the observations took place over 24 days (12 days in 2014; 12 days in 2015) of which eight were cloudy or windy days and only 2 days were partially rainy from 08:00 AM to 06:00 PM without interruption, and for 35 different flowers overall. It is important to note that during the spring in this region, the sun rises at ~6:00 AM, but due to the landscape and vegetation, it touches the *P. secreta* plants at this site only after 8:00 AM and remains until 6:00 PM.

The behaviour of visitors was directly observed, and photographs and videos were taken using a Nikon D3200 SLR camera with a Nikon DX AF-S Nikkor 18-55 mm microlens (Nikon Co.) positioned 3 m from the flowers to reduce any interference due to observer presence. We recorded the number and taxonomic group of visitors and their behaviour during the visit, the landing site on the flower, contact with pollen, the position of pollen grains on the pollinator body, the ability to touch the stigma, visit duration, floral resource type collected and number of visited flowers.

We recorded the total number of visitors per individual flower and the number of flowers visited by each kind of visitor. The frequency of visits was analysed by dividing the number of visits made by each visitor by the total number of visits or pollinations during all observations per year.

We classified animals as visitors or pollinators based on their behaviour and likelihood to conduct effective pollination. We considered as legitimate pollinators of *P. secreta* only those floral visitors that had contacted the anthers and stigma for long enough to transfer the pollen. Floral visitors were divided into four functional groups according to Fenster *et al.* (2004), namely, long-tongued bees, short-tongued bees, hummingbirds and hawkmoths. Insects considered as potential pollinators were collected and preserved in 70 % ethanol for taxonomic identification and deposited in the Science and Technology Museum, Pontifical Catholic University of Rio Grande do Sul (Porto Alegre, Rio Grande do Sul, Brazil).

## Results

### Floral biology

*Petunia secreta* bloomed from September to December in both years. In the field, flower opening occurred only during the daytime; the flowers remained open for ~2 days if pollen contacted the stigma, but became senescent after 4 days in the absence of pollination. Anther dehiscence took place simultaneously with flower opening.

### Floral traits

**Figure 2.**
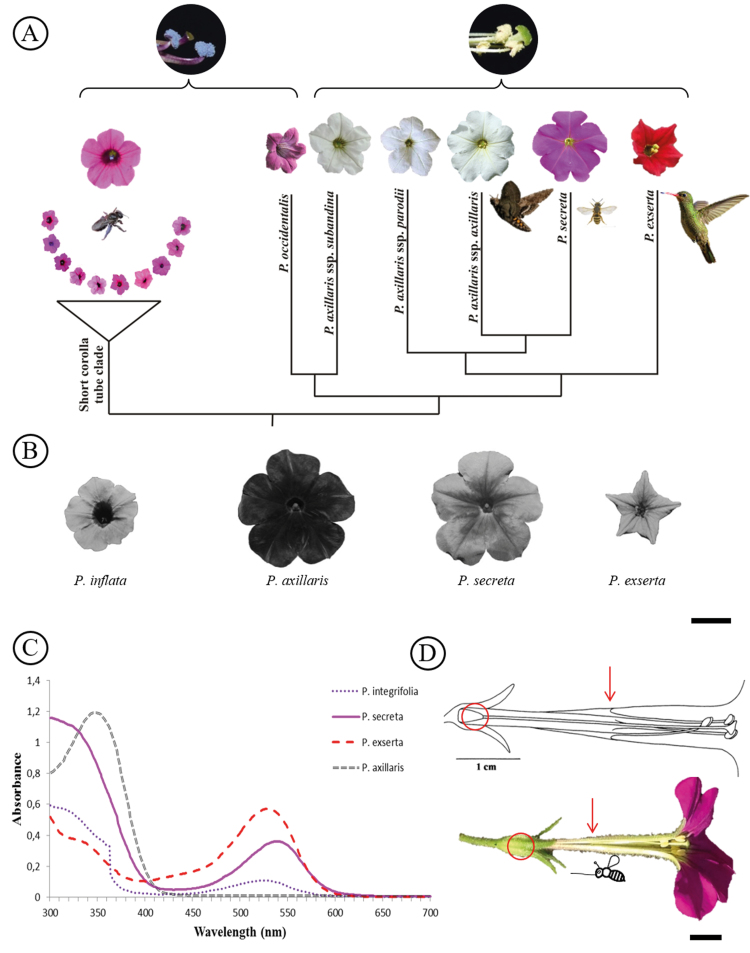
Pollinator attraction cues: comparison among *Petunia* species. A, Phylogenetic tree of the *Petunia* genus highlighting the relationships between corolla and pollen colours and pollinators (adapted from Reck-Kortmann *et al.* 2014); B, UV light responses in detached flowers of four *Petunia* species (UV absorbing = dark flowers; UV reflecting = light flowers). A flower of *P. inflata* represents the short corolla tube clade; C, pigment components of petals in *Petunia* species. Different peaks represent different pigment chemical classes according to the wavelength range, and the lines correspond to different species (see legend); D, *P. secreta* linear flower design (adapted from Stehmann *et al.* 2009) and nectar position. The circle corresponds to the location of nectar secretion, and the arrow indicates the point at which anther filaments start to fuse to the floral tube and form the compartment for the style. Bar = 1 cm.

In *P. secreta,* the nectar was secreted at the base of the corolla (Fig. 2D), the total volume ranged from 4 to 20 μL per flower (mean 8 μL) and the total sugar concentration varied from 16 to 26 % per flower (mean 21.5 %) across the 20 flowers measured **[see****Supporting Information—Table S2****]**. The stigma receptivity tests revealed that the stigma surface was active during all stages, suggesting that the *P. secreta* stigma is receptive before flower opening until withering of the petals.

Flowers of the four *Petunia* species differed in appearance under visible (Fig. 2A) and UV light (Fig. 2B). UV light revealed that, as expected for flowers pollinated by moths, *P. axillaris* petals absorbed UV light (dark colour); the petals of *P. inflata* and *P. secreta* reflected UV light (light colour), a trait that is associated with bee-pollinated flowers. The petals of bird-pollinated *P. exserta* also reflected UV light (light colour).

*Petunia secreta*, *P. axillaris*, *P. exserta* and *P. integrifolia* (Fig. 2C) showed the presence of flavonols within a 302–340 nm wavelength range, with *P. axillaris* and *P. secreta* exhibiting higher values of absorbance at 1.19 and 1.15, respectively, whereas *P. exserta* and *P. integrifolia* demonstrated values of 0.38 and 0.58, respectively. Anthocyanin peaks appeared in *P. exserta*, *P. secreta* and *P. integrifolia* within a 524–538 nm wavelength range; absorbance values were species-specific at 0.56, 0.36 and 0.10, respectively. *Petunia axillaris* did not present any peaks within the anthocyanin range.

### Pollen aroma compounds

**Figure 3.**
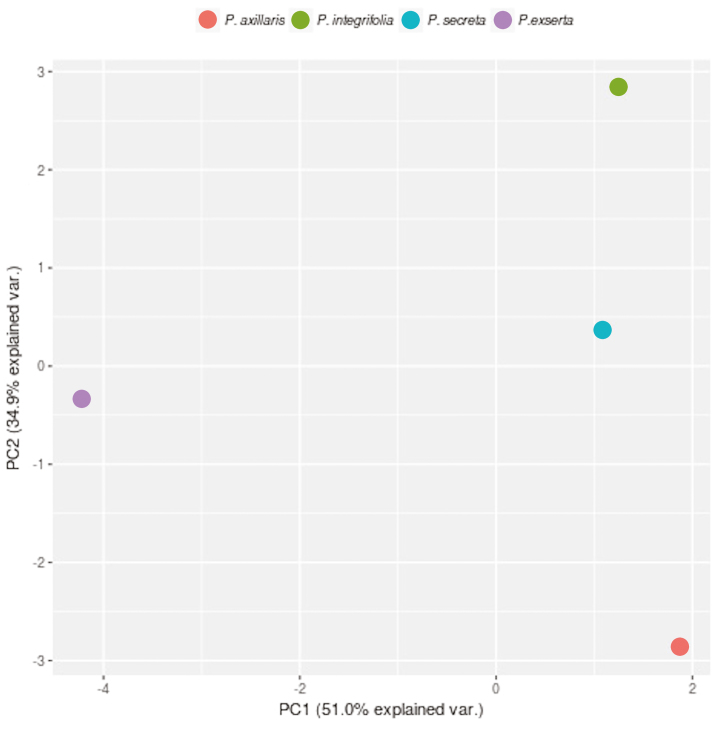
Principal component analysis based on pollen-emitted scents for four *Petunia* species.

Gas chromatographic measurement from pollen scents revealed 63 biologically active compounds, mainly aliphatic compounds (35), benzenoids (16), cyclic compounds (5), terpenoids (4), organic compounds (1) and nitrogen compounds (2). Only three compounds (1-butanol, 3-methyl, 2-butanone, 3-hydroxy and phenylethyl alcohol) were found in all four species, indicating that the scent profiles differ among the four *Petunia* species **[see****Supporting Information—Table S3****]**. *Petunia exserta* presented the highest number of exclusive compounds (23), whereas *P. secreta* showed the lowest number of compounds (only one exclusive), and the greatest similarity in composition was observed between *P. integrifolia* and *P. secreta*. *Petunia secreta* and *P. axillaris* were quantitatively most similar, and the volatile emissions of *P. exserta* differed the most from those of the other species (Fig. 3).

Thirty-one compounds were detected in the pollen of *P. axillaris* with methylbenzoate, 2,3-butanediol, 3,7-dimethyldecane and phenylethyl alcohol being the major compounds. *Petunia integrifolia* pollen contained 12 compounds, with the most abundant being isobutyl phthalate, *cis*-caryophyllene, 1-butanol, 3-methyl and ethanol. The major constituents of the *P. exserta* pollen aroma were toluene, 2-pyrrolidinemethanol, 1-methyl 3-amino-5-tert-butylpyrazole and 1-octanol, with a further 34 compounds being detected. Finally, *P. secreta* presented 15 VOCs in pollen, with methylbenzoate, 2,3-butanediol, ethanol, 1-butanol, 3-methyl and isoeugenol being the most frequent pollen aroma compounds **[see****Supporting Information—Table S3****]**. *Petunia secreta* shared 14 of its pollen VOCs with at least one of the three other analysed species, of which 12 were previously reported to elicit positive behaviour in bees, four were reported to attract hawkmoths and two have not been evaluated to date (Table 1).

**Table 1.**
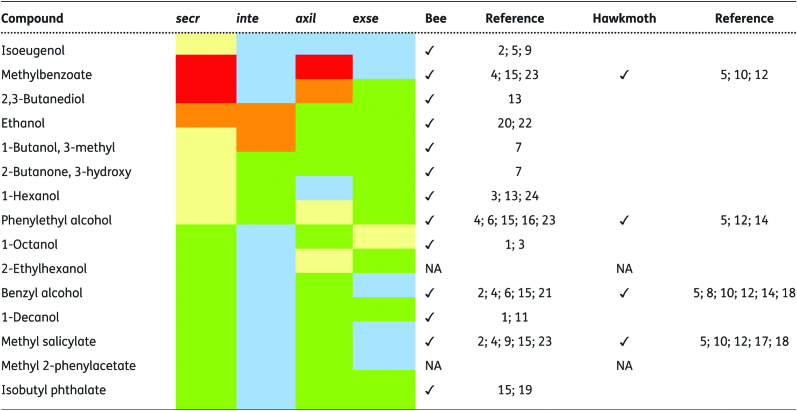
Chemical composition of pollen scent in *Petunia secreta* compared to the other three *Petunia* species. Colours reflect maximum emission (% in relation to total emission): blue (0%); green (<5%); yellow (>5% and <10%); orange (>10% and <20%); red (>20%). We considered a floral scent compound attractive when it was reported in the literature as eliciting positive bee and/or hawkmoth behaviour. secr – *Petunia secreta*; inte – *P. integrifolia*; axil – *P. axillaris*; exse – *P. exserta*; ✓– compound described as attractive for; NA – not available; References: 1 - Braunschmid *et al.,* 2017; 2 - Carril, 2014; 3 - Cordeiro *et al.*, 2017; 4 - Dötterl and Vereecken, 2010; 5 - Dudareva and Pichersky, 2006; 6 - Filella *et al.*, 2011; 7 - Goodrich *et al.*, 2006; 8 - Haverkamp *et al.*, 2016; 9 - Hetherington-Rauth and Ramírez, 2016; 10 - Hoballah *et al.*, 2007; 11 - Johnson *et al.*, 2005; 12 - Knudsen and Tollsten, 1993; 13 - Knudsen *et al.*, 2006; 14 - Levin *et al.*, 2001; 15 - Pham-Delègue *et al.*, 1992; 16 - Raguso, 2004; 17 – Raguso and Light, 2003; 18 – Raguso *et al.*, 1996; 19 - Teichert *et al.*, 2012; 20 - Vega *et al.*, 2009; 21 - Wadhams *et al.*, 1994; 22 - Wiens *et al.*, 2008; 23 - Williams and Whitten, 1983; 24 - Wright *et al.*, 2008.

### Pollinator and visitor observations

Visitors of three different functional groups were observed on flowers of *P. secreta* (Table 2) during the 225 h of observations **[see****Supporting Information—Table S4****]**. Bee species of two genera with short tongues, one genus of long-tongued bees and one unidentified hummingbird species were recorded foraging and visiting *P. secreta*. We counted 51 visits in total of which 39 (76 %) were by species of Halictidae; *Pseudagapostemon* sp. bees were the most frequent visitors. Most pollinator visits occurred during the afternoon from 1:00 PM to 5:00 PM, with the peak occurring between 1:00 PM and 3:00 PM. There was a low frequency of visits to *P. secreta* during the first few hours of the day **[see****Supporting Information—Fig. S1****]**.

**Table 2.** Visitation and pollination frequency of different functional groups as observed in *Petunia secreta* per year. ND—not determined; % visitation corresponds to the frequency of views without pollen transfer to stigma; % pollination corresponds to the frequency visits with pollen transfer to stigma; – no views or pollen not transferred.

Floral visitors	Classification	Functional group	2014 (120 h)		2015 (105 h)	
			% Visitation	% Pollination	% Visitation	% Pollination
*Pseudagapostemun* sp.	Halictidae	Short-tongued bee	81	**100**	–	**100**
*Lanthanomelissa clementis*	Apidae	Short-tongued bee	19	–	–	–
*Xylocopa* sp.	Xylocopinae	Long-tongued bee	–	–	45.5	
Unidentified bee	Hymenoptera	ND	–	–	9	–
Unidentified bird	Trochilidae	Hummingbird	–	–	45.5	–

Bees belonging to the *Pseudagapostemon* genus (Fig. 1B) landed directly on the anthers and collected pollen exclusively. These individuals approached the flowers, flew away and approached again several times until they landed. We counted 38 visits, most of them occurring in 2015, in the same season in which we observed more flowers of *P. secreta*. Individuals of *Pseudagapostemon* sp. displayed a behaviour and body structure that fulfilled all our criteria for consideration as a legitimate pollinator of *P. secreta*. We observed 22 visits of *Pseudagapostemon* sp. individuals that landed directly on the reproductive structures (Fig. 1C), with the front legs scraping the anthers and transferring pollen to the scopa in the abdomen (dense set of hair or bristles specialized for pollen adherence) and to the hind tibia **[see****Supporting Information—Movie S1****]**, always positioned on the flower with the abdomen and legs in front of the stigma. These individuals took, on average, <2 min to collect pollen.

*Pseudagapostemon* sp. individuals were observed on *P. secreta* flowers only when pollen was present; therefore, each flower received a maximum of two visits. In the presence of pollen, bees removed all pollen, and in its absence, bees did not land. Differences in anther height (Fig. 1D) influenced bee behaviour during pollen collection, making the insect stand in different directions and slip on the stigma surface to completely remove the pollen. There was no standard time of day for visits by *Pseudagapostemon* to *P. secreta* flowers; visits were spread from 10:30 AM to 6:00 PM. Visits occurred on sunny days, and none occurred on rainy, windy or cloudy days **[see****Supporting Information—Table S4****]**.

Four male individuals of *Lanthanomelissa clementis* were seen only once at dusk, in a group using the flower as a dormitory, and remained inside the flower until the following morning. One individual of *Xylocopa* sp. that was seen on 1 day in October 2015 visited the flowers several times, cut out a piece of the corolla and took it away. The unidentified hummingbirds were observed once in October 2015 and four times in November 2015; on each visit, the bird introduced its bill into the flower on average for ~3 s, and restricted itself to one flower per visit. We were not able to take photos that would allow identification of the species of hummingbirds, and we were also not able to verify effective pollination by these birds. We think the birds are unlikely to transfer much pollen, which would be confined to the beak and not the feathers, and due to the flower morphology, which hides the anthers inside the tube. However, it is possible that the birds shake the flower and pollen could be deposited on the stigma, promoting self-pollination by a secondary pollinator.

## Discussion

### Putative pollinator as suggested by morphological traits

We investigated the floral traits and the plant–pollinator interactions of *P. secreta*. *Petunia secreta* presents a set of floral features such as pink corolla, diluted nectar and volatiles in pollen that distinguish it from the other *Petunia* species. We found that *P. secreta* is mainly pollinated by Halictidae, a functional group of short-tongued bees. We cannot say that other insects or birds never promote pollination. The pollen scent and flower colour are likely adaptations that attract bees. However, the narrowness of the corolla tube and the nectar characteristics seem to be adaptations for other pollinators, possibly hummingbirds or some kind of Lepidoptera, although we rarely saw hummingbirds and never saw any Lepidoptera visiting *P. secreta*.

Interspecific differences in the UV-light floral response are found among the *Petunia* species that are indicative of their pollination syndromes*. Petunia secreta* has a pink corolla that reflects UV light, traits mainly present in bee-pollinated species (Papiorek *et al.* 2016) and appears to reflect the ancestral state of the *Petunia* genus (Reck-Kortmann *et al.* 2014), represented here by *P. inflata*. However, *P. secreta* does not have all the typical floral features known for the bee-pollination syndrome, such as unscented flowers, blue pollen, a low volume of nectar and a wide and short corolla tube. Conversely, *P. secreta* shares several traits with *P. axillaris*, such as the long and narrow corolla morphology that limits nectar access by large insects, similar amounts of flavonoids in flowers, moderate nectar resources and odour emitted from its yellow pollen comprising compounds that have previously been described as attractive to diverse insect species. Of note, *P. secreta* does not emit a floral scent at dusk, which is the main characteristic that attracts hawkmoths in *P. axillaris* (Venail *et al.* 2010; Klahre *et al.* 2011).

The *P*. *secreta* reflectance peak spectrum suggests that the corolla is attractive to bees. However, petals of *P. secreta* are also within the range of vision of hummingbirds, which perceive colour over wavelengths ranging from 300 to 600 nm (Cronk and Ojeda 2008). Nevertheless, the visual display differs by corolla colour and reflectance among the species analysed here, and suggests that *P. inflata*, *P. secreta* and *P. exserta* are visually more adapted to diurnal pollinators and *P. axillaris* to nocturnal pollinators. The contrast in floral colour suggests that *P. secreta* and *P. axillaris* are adapted to different pollinator assemblages, and the similarity in their pollen odours possibly reflects their shared evolutionary relationships (Reck-Kortmann *et al.* 2014). Thus, the pink colour could be associated with an increase in detection by the bees and with a decrease in detection by nocturnal moths (Venail *et al.* 2010).

Nectar volume varies among *Petunia* species. The average volume and sugar concentration of *P. secreta* nectar are lower than those observed in *P. axillaris* subsp. *axillaris* (Gleiser *et al.* 2014), but the volume is much higher than that reported for *P. integrifolia* (Gübitz *et al.* 2009). This result shows that *P. secreta* produces a modest amount of nectar; despite this, the nectar does not act as a reward for the most frequent pollinator (*Pseudagapostemon* sp.) but is occasionally used by hummingbirds.

The sugar concentration in nectar from *P. secreta* matches what has been proposed for bird-pollinated flowers (Baker 1975; Proctor *et al.* 1996). Birds that effectively introduce their bill into flowers can promote pollen transference when visiting flowers searching for available nectar (Maruyama *et al.* 2013). The characteristics of nectar from *P. exserta* flowers are not known, but they present several traits related to hummingbird pollination such as their bright red corollas (Gübitz *et al.* 2009), backward-folding petal limbs and reproductive structures exerted from the corolla, which improves contact with the bird’s head and facilitates pollen transfer (Lorenz-Lemke *et al.* 2006; Stehmann *et al.* 2009). *Petunia secreta* lacks all these morphological traits of *P. exserta.*

The colour and scent are equally important to hawkmoths in foraging decisions among flowers with different morphologies (Glover 2011). Flowers of *P. axillaris* support this observation (Venail *et al.* 2010; Klahre *et al.* 2011) despite possessing a nectar volume and sugar concentration close to the optimal amounts reported not only for hawkmoths (Gleiser *et al.* 2014) but also for several types of bees (Kim *et al.* 2011). The loss of flower odour and gain of visible colour in *P. exserta* is likely related to the greater trend towards bird pollination compared to *P. axillaris* (Kessler *et al.* 2013; Amrad *et al.* 2016), and we think this could also be the case in *P. secreta*. Some shared traits present in all long corolla tube species could correspond to shared ancestral features and may not be related to the most important pollinator.

### The role of pollen scent in relation to pollinator affinities

There is little information on pollen scents for most plants. However, it is known that bees are able to discriminate between pollen odours in biologically realistic concentrations, which suggests that the pollen odour may attract these pollinators (Cook *et al.* 2005; Ruedenauer *et al.* 2015).

The VOCs described here from *Petunia* pollen are generally present in flower bouquets (Knudsen *et al.* 2006), and some of them are among the most frequently observed (Knudsen *et al.* 1993). Each *Petunia* species emits a characteristic mixture of volatiles with distinct compounds and different total amounts that are compatible with the pollination syndrome.

We found that plant–pollinator interactions in *P. secreta* cannot be interpreted as a bee-pollination syndrome based only on UV-light response and corolla colour, and pollen volatiles can have an important effect on the legitimate pollinator, *Pseudagapostemum* sp., since VOCs in this species are associated with responses by bee antennae in other angiosperms that allow the bees to detect pollen from a distance before landing (Dötterl *et al.* 2005; Dötterl and Vereecken 2010). Indeed, different sets of volatile pollen compounds are shared between *P. secreta* and *P. integrifolia,* both of which display a corolla colour and UV reflectance related to bee pollination, and some compounds are found at high levels that are unusual in floral aroma and are associated with bee attraction (Goodrich *et al.* 2006).

In *P. axillaris,* the most abundant compound in the pollen scent profile is one of the three most frequent endogenous VOCs (Negre *et al.* 2003), and several others associated with bee pollination are also present in similar proportions to those observed in *P. secreta* pollen. The similarity between these two species may be explained based on their evolutionary proximity and because diurnal secondary pollinators in *P. axillaris* were observed (Gübitz *et al.* 2009).

The pollen scent profile in *P. exserta* presents the highest number of exclusive compounds, many of which are related to defence against herbivores. Plants with exposed pollen like *P. exserta* produce anti-herbivore deterrents in pollen (Dafni *et al.* 2000) and have specific floral bouquets to deter florivores and nectar robbers and simultaneously attract pollinators and antagonists (Schiestl *et al.* 2014; Kessler *et al.* 2015).

### Legitimate pollinators of *Petunia secreta*

Based on general floral colour and shape, *P. secreta* was described as a bee-pollinated species (Stehmann and Semir 2005), and our findings support this assertion.

*Pseudagapostemon* sp. bees can be attracted to *P. secreta* by corolla colour and UV reflectance, but pollen volatiles also appear to play a role because bees land only on flowers with at least one intact anther. The pollen aroma may be involved in specific pollinator attraction at short distances. *Pseudagapostemon* sp. behaviour is consistent with the view that certain pollen compounds constitute a stimulus to bees landing. The critical nature of the floral scent in the foraging behaviour of host-specialized solitary bees has been demonstrated in honeybees that associate scent and pollen (Arenas and Farina 2012).

Although *P. secreta* flowers secrete nectar, bees cannot reach the bottom of the corolla tube to collect it. Field observations (data not shown) and previously published measurements (Stehmann and Semir 2005; Turchetto *et al.* 2016) indicate that the distance between the point at which the filaments fuse to the corolla and the deeper portion of the tube where the nectar accumulates is small (ca. 2 cm), and the tube along this region is slender (ca. 2–4 mm in diameter). These measurements suggest that it is impossible for individuals of *Pseudagapostemon* sp. to gather the nectar in contrast to *Callonychium* individuals that can obtain nectar from flowers of *P. integrifolia* (Wittmann *et al.* 1990). The body length of *Pseudagapostemon* sp. is ca. 5–11 mm (Michener 2007), but the length of the proboscis is not known; however, even if the tongue is as long as the body, it would still not cover the distance of 20 mm required to reach the nectar in *P. secreta*. Furthermore, *Pseudagapostemon* sp. individuals did not act as nectar collectors in any of the observed visits.

Bees visited the flowers, but each visit was restricted to a single flower, and the asynchronous mass flowering in *P. secreta* may reduce the frequency of geitonogamy since this species produces only one flower per time per individual and just a few individuals make up the plant patches at the studied site (Turchetto *et al.* 2016). However, *Pseudogapostemon* sp. appeared to be responsible for the most pollination events, and its pollen collection behaviour likely promotes a high frequency of self-pollination (D. M. Rodrigues *et al.*, unpubl. data). *Petunia secreta* is self-compatible (Rodrigues *et al.* 2018) and shows a high genetic diversity compared to other congeneric species (Turchetto *et al.* 2016), probably due to secondary pollinators that promote cross-fertilization.

We found that short-tongued bees mainly pollinate *P. secreta* plants; however, the field observations and some floral cues do not allow us to rule out hummingbirds and other insects as occasional pollinators.

### Do we truly understand pollination syndromes in *Petunia* as much as we suppose?

In an evolutionary context, our findings suggest that the interaction with *Pseudagapostemon* sp. bees has minimized the nectar volume and concentration of *P. secreta* compared to other sympatric *Petunia* species, while maximizing pollen scent emission, corolla colour and UV-light reflectance to improve the attraction of short-tongued bees, all characteristics that are ancestral conditions of the genus. However, different functional groups of pollinators can play a role in the reproductive success of *P. secreta*. Oligolectic bees were observed most frequently pollinating *P. secreta* plants, but hummingbird pollination also seems to occur.

Evolutionary shifts from one pollination syndrome to another often involve particular flower colour transitions (Wessinger and Rausher 2012). The major determinant of flower colour variation between *P. integrifolia* and *P. axillaris* that has caused major shifts in pollination is the *ANTHOCYANIN2* gene (Hoballah *et al.* 2007), with gene inactivation promoting the change in corolla colour from pink to white. Moreover, traits such as scent emission, flower architecture and rewards can be clustered and allow rapid switching between pollination syndromes in response to changes in pollinator availability (Hermann *et al.* 2013), as in *P. axillaris* and *P. exserta*.

Studies focusing on the traits involved in host finding by oligolectic bees concluded that visual and olfactory cues are used when bees search for food (Burger *et al.* 2010; Milet-Pinheiro *et al.* 2012; Carvalho *et al.* 2014). *Petunia secreta* may be visited by different functional groups, but the relative selective pressures that they exert may be different. The corolla colour, as well as the lack of corolla scent at dusk and a low sugar concentration in nectar, can prevent visits by nocturnal pollinators, especially hawkmoths. The pollen odour may attract some pollinators and possibly be inconspicuous to other insects. The absorbance spectrum in petals of *P. secreta* suggests the ability to attract bees; however, flowers of *P. secreta* can be easily detected by hummingbirds and it is known that species with non-red flowers are occasionally hummingbird-pollinated, especially at sites where their preferential pollinators are found at low densities (Cronk and Ojeda 2008).

Flower and pollinator features contribute to restricting pollination to individuals of the same species and enhance reproductive isolation in a variety of plant species (Scopece *et al.* 2014; Breitkopf *et al.* 2015). It can be argued that classifying flowers as belong to a single specialized pollination syndrome may mask the importance of ‘secondary’ or ‘tertiary’ pollinators as drivers of particular floral traits (Queiroz *et al.* 2015; Cronk and Yang 2016) especially in *Petunia*, because in this genus we can observe natural hybrids between *P. axillaris* and *P. exserta* (Segatto *et al.* 2014; Turchetto *et al.* 2015b) that have different pollinators: hawkmoths and bees visit and pollinate *P. axillaris* (Gübitz *et al.* 2009) and *P. secreta* presents different putative pollen vectors. All these species occur in the same geographical area.

Moreover, the validity of pollination syndromes has been widely questioned (Rosas-Guerrero *et al.* 2014; Gong *et al.* 2015). Sympatric species mainly depend on specific floral traits to establish relatively strict but not absolute pollinator specificity, and pollinator sharing could be rather common (Wang *et al.* 2016) and would explain the interrelationships we observed among the *Petunia* species. When adaptation to a slightly effective pollinator requires minimum loss of fitness compared to a more effective pollinator, plant species may exhibit specialized traits for secondary pollinators (Aigner 2001).

## Conclusions

The variation in colour, nectar and pollen scent of different co-occurring species of *Petunia* can provide information on the specific signals that guide pollinators and may contribute to reproductive isolation. This is a preliminary report on variation in pollen scent from different wild *Petunia* species and the first field observations of the pollination ecology of *P. secreta*. Combined, these data suggest that *P. secreta* exhibits a set of traits that enable these plants to be effectively pollinated by solitary bees despite preserving some shared traits with its cousins that are pollinated by other kinds of animals. Moreover, pollen scents may have evolved in conjunction with the sensory capabilities of different visitors rather than the specific group of pollinators seen to visit representative *Petunia* species with documented pollination syndromes. A number of these characteristics, especially the colour of petals and abundant amounts of some compounds in pollen, represent a reversion to the ancestral condition in the *Petunia* genus since they are shared with other bee-pollinated species. Despite this, we cannot rule out the possibility that other floral traits (pink long tubular flowers, and nectar volume and sugar concentration) could attract other functional groups of pollinators (probably hummingbirds) and could constitute an evolutionary shift in the pollination system in progress.

The collection, isolation, identification and bioassay of the pollinator attractants from *Petunia* deserve further attention in order to investigate the potential interaction between olfactory and other signals in *Petunia* species and detect which are the most important compounds in mutualistic interactions. Additionally, olfactory experiments are required to test how the bee’s behaviour differs between unique blends and the overall quantity of volatile emissions.

## Supporting Information

The following additional information is available in the online version of this article—

**Table S1.** Biological sources for different analyses and comparisons.

**Table S2.** Nectar volume and sugar concentration.

**Table S3.** List of pollen volatile organic compounds in four *Petunia* species.

**Table S4.** Visitor and pollinator records per year and flower.

**Figure S1.** Frequency of visitations in flowers of *Petunia secreta.* Pse: *Pseudagapostemon* sp. (Halictidae); Lan: *Lanthanomelissa clementis* (Apidae); Hum: unidentified hummingbird (Trochilidae); Xylo: *Xylocopa* sp. (Apidae); Ubee: Unidentified bee (Apidae).

**Movie S1.** *Pseudagapostemon* sp.: the legitimate pollinator of *Petunia secreta*.

Supplementary Material

Supplementary Video

## Sources of Funding

This work was supported by the Conselho Nacional de Desenvolvimento Científico e Tecnológico (CNPq), Coordenação de Aperfeiçoamento de Pessoal de Nível Superior (CAPES), Fundação Grupo O Boticário de Proteção à Natureza and Programa de Pós-Graduação em Botânica da Universidade Federal do Rio Grande do Sul (PPGBot-UFRGS).

## Contributions by the Authors

D.M.R. and L.B.F. planned, designed and led the project; D.M.R., L.C.-V., C.T. and R.A.J. conducted the experiments, ran the analyses; D.M.R., L.C.-V. and L.B.F. wrote most of the text; C.K. and L.B.F. provided reagents and equipment to develop the experiments. All authors contributed in the preparation of the study and have commented on and approved the final manuscript.

## Conflict of Interest

None declared.
