# Supplementary material for: Do we truly understand pollination syndromes in Petunia as much as we suppose?
Source: AoB Plants. 2018 Oct 1;10(5):ply057. doi: 10.1093/aobpla/ply057 (PMC6202611; doi:10.1093/aobpla/ply057)
Supplement: Supplementary Material [file ply057_suppl_supplementary_material.pdf]

Table S1. Biological sources for different analyses and comparisons

Analyses	Species	Sources	Geographic coordinates	Voucher
Nectar evaluation and stigmatic receptivity	*P. secreta*	Collected in nature; Caçapava do Sul, Rio Grande do Sul, Brazil	30°32’45” S 53°33’00” W	BHCB76025*
UV-light response	*P. secreta*	Collected in nature; Caçapava do Sul, Rio Grande do Sul, Brazil	30°32’45” S 53°33’00” W	BHCB76025
	P. axillaris N	Cultivated; Rostock Botanical Garden (Germany)		
	P. exserta	Cultivated; R. Koes (University of Amsterdam, the Netherlands)		
	P. inflata S6	Cultivated; R. J. Griesbach (Beltsville, USA)		
Pigments estimate and pollen scent composition	*P. secreta*	Collected in nature; Caçapava do Sul, Rio Grande do Sul, Brazil	30°32’45” S 53°33’00” W	BHCB76025
	P. axillaris	Collected in nature; Caçapava do Sul, Rio Grande do Sul, Brazil	30°89’63” S 53°42’08” W	ICN185145#
	P. exserta	Collected in nature; Caçapava do Sul, Rio Grande do Sul, Brazil	30°83’69” S 53°50’41” W	ICN185146
	P. integrifolia	Collected in nature; Caçapava do Sul, Rio Grande do Sul, Brazil	30°51’22” S 53°49’14” W	ICN181349

*Herbarium of Universidade Federal de Minas Gerais, Belo Horizonte, MG, Brazil;
#Herbarium of Universidade Federal do Rio Grande do Sul, Porto Alegre, RS, Brazil.

Table S2. Nectar volume and sugar concentration per individual of *Petunia secreta* from Pedra do Segredo. (s.d.) standard deviation

Individual code	Flower number	Nectar volume (μL)	Sugar concentration (%)
59A	1	4	16
59A	2	8	22
59A	3	4	23
59A	4	8	19
59A	5	4	21
Mean (s.d.)		5.6 (2.2)	20.2 (2.8)
59B	1	20	25
59B	2	10	25
59B	3	12	22
59B	4	10	24
59B	5	12	26
Mean (s.d.)		12.8 (4.1)	24.4 (1.5)
59C	1	9	23
59C	2	15	20
59C	3	4	20
59C	4	6	21
59C	5	4	20
Mean (s.d.)		7.6 (4.6)	20.8 (1.3)
59D	1	10	24
59D	2	8	20
59D	3	6	21
59D	4	8	21
59D	5	4	23
Mean (s.d.)		7.6 (2.3)	21.8 (1.6)
**Mean (s.d.)**		**8 (4.2)**	**21.5 (2.4)**

Table S3. List of pollen volatile organic compounds (VOCs) given as the relative amount (%) of pollen-emitted scents by four *Petunia* species, 10 individuals per species.

Compound	*P. secreta*		*P. integrifolia*		*P. axillaris*		*P. exserta*	
	RT	%	RT	%	RT	%	RT	%
**Aliphatic**								
1-Butanol, 3-methyl	7.62	7.98	7.52	12.29	7.61	3.67	7.61	0.59
2-Butanone, 3-hydroxy	9.09	6.50	8.99	4.90	9.09	3.15	9.09	0.89
2-Ethylhexanol	12.50	1.79			12.52	5.42	12.54	3.00
2,3-Butanediol	13.30	21.03			13.60	13.50	13.61	3.12
Ethanol	3.11	10.21	3.05	11.48	3.11	2.22		
1-Octanol	13.60	3.89			13.59	3.14	13.60	5.67
1-Decanol	16.60	1.56			16.60	1.11	16.62	1.78
1-Hexanol	10.20	5.70	10.13	3.11			10.23	3.43
2-Hexenal					7.85	0.29	7.85	0.50
Hexadecane					8.70	1.20	9.30	0.54
1-Octen-3-ol			11.79	5.97	11.88	2.06		
3,7-dimethyldecane					5.46	7.08		
Butanoic acid					7.35	1.14		
Dodecane					7.49	1.56		
2,6-dimethylnonane					4.55	0.56		
4,6-dimethyl-dodecane					8.34	2.07		
3-Hexenol					10.78	0.57		
3-methyl-1,5-pentanediol					11.58	0.18		
2,6,11-trimethyldodecane					11.69	0.57		
Tetradecane					11.77	1.01		
Heptadecane					12.67	1.15		
Hexanal							5.36	0.82
Undecane							5.65	0.66
Heptanal							7.25	0.41
6-ethyl-2-methyldecane							7.49	0.96
Octanal							9.16	0.93
Nonanal							10.99	2.85
2-Butoxyethanol							11.09	0.73
Decanal							12.74	3.24

Nonanol							15.14	0.80
2-Ethyl-2-methyl-1,3-propanediol							14.12	0.48
Propanoic acid, 2-methyl-, 1-(1,1-dimethylethyl)-2-methyl-1,3-propanediyl ester							18.22	2.21
Propanoic acid, 2-methyl-, 2,2-dimethyl-1-(2-hydroxy-1-methylethyl)propyl ester							18.33	0.30
2-ethylhexanoic acid							19.26	0.75
Octanoic Acid							20.66	0.42
**Benzenoid**								
Toluene					4.63	0.83	4.60	21.39
Methyl 2-Phenylacetate	16.93	0.76			16.68	1.21		
Methyl Salicylate	16.93	1.35			16.93	1.77		
Benzyl Alcohol	18.25	1.73			18.25	2.86		
Diethyl Phthalate					24.16	0.67	24.18	1.64
Methylbenzoate	14.69	22.99			14.69	26.46		
Phenylethyl Alcohol	18.70	5.52	18.60	3.17	18.70	6.85	18.72	0.42
Isobutyl phthalate	25.93	1.03			25.93	1.52	25.95	2.04
Benzyl nitrile					18.98	0.35		
Acetyleugenol					23.94	3.87		
Acetic acid							12.19	1.03
Isoeugenol	23.94	7.90						
p-octyloxybenzoic acid			23.27	9.47				
Dinonyl phthalate			24.04	2.84				
Isobutyl phthalate			25.82	25.29				
Phenethylbenzoate			27.62	3.93				
**Cyclic**								
3-Cyclohexene-1-ethanol					15.66	1.79		
Menthol							14.88	0.87
α-Terpineol							15.74	0.42
D-carvone								0.75
Azulene							16.46	2.27
**Terpenoids**								
Neoalloocimene			22.12	4.77				
6-Methyl-5-hepten-2-one								0.62
Cis-caryophyllene			21.83	12.73				
**Irregular terpenes**								
Nerylacetone								1.28
**Nitrogene**								
2-Pyrrolidinemethanol, 1-methyl							12.80	20.93

3-Amino-5-tert-butylpyrazole	9.50
**Organic**	
Hexanedioic acid	1.60

RT – Kováts retention index (according: UPAC. Compendium of Chemical Terminology, 2nd ed. A.D. McNaught and A. Wilkinson. Blackwell Scientific Publications, Oxford, 1997).

Table S4. Records of visitors and pollinators for *Petunia secreta* from Pedra do Segredo (30º 32' 45.9'' S 53º 33' 00.9'' W) carried out in two flowering seasons.

Year	Day	Whether	Flower(1)	Time (h)/day	Visitor(2)	N(3)	Mean Time (s)(4)	Pollinator(5)	N(3)	Mean Time (s)(4)
2014	Sept 26	Windy	1	10	-			-		
	Sept 27	Cloudy	2	10	-			-		
	Oct 08	Sunny	3	10	Pse	10	60	Pse	1	125
			4		Lan	4	ND	-		
	Oct 09	Sunny	4	10	Lan	4	ND	-		
			5		Pse	3	60	Pse	2	62
	Oct 10	Windy	6	10	-			-		
	Oct 23	Sunny	7	10	Pse	2	50	Pse	2	120
	Oct 24	Windy	8	10	-			-		
	Oct 25	Cloudy	9	10	-			-		
	Nov 04	Sunny	10	10	-			Pse	1	110
	Nov 06	Sunny	11	10	-			Pse	1	180
	Nov 25	Windy	12	10	-			-		
	Nov 26	Sunny	13	10	Pse	2	60	Pse	1	120
2015	Oct 06	Windy	14	10	-			-		
	Oct 07	Windy	15	10	-			-		
	Oct 08	Sunny	16	10	-			Pse	1	150
	Oct 21	Sunny	17	10	-			Pse	1	120
	Oct 22	Sunny	18	10	-			Pse	1	160
			19		Hum	1	3	-		
			20		Xyl	5	10	-		
	Nov 03	Sunny/Windy	21	10	-			Pse	1	90
	Nov 04	Cloudy/Rainy	22	2.5	-			-		
	Nov 05	Cloudy/Rainy	23	2.5	-			-		
	Nov 06	Sunny	24	10	Hum	1	5	Pse	2	102
			25		Ubee	1	8	-		
	Nov 07	Sunny	26	10	-			Pse	1	53
			27		-			Pse	1	180
			28		-			Pse	1	60

	Nov 23	Sunny	29	10	-			Pse	1	120
	Nov 25	Sunny	30	10	-			Pse	1	120
			31		-			Pse	1	180
			32		-			Pse	1	120
			33		-			Pse	1	40
			34		Hum	1	3	-		
			35		Hum	2	3	-		
**Total/ mean**	**24**		**35**	**225**	**5***	**36#**	**8$**	**1***	**22#**	**101$**

(1)Tag ID for different flowers; (2)All animals that foraged *P. secreta* flowers; (3)Number of visiting or pollination events for same or different individuals; (4)Mean time spent considering all visiting or pollination events; (5)All animals that effectively had contacted the reproductive organs (pistil and stamens) of *P. secreta* flowers at suitable time; *Number of different visitor or pollinator groups; #Total number of visits or pollinations; $Mean time per visit or pollination considering all events per class; Pse – *Pseudagapostemon* sp.; Lan - *Lanthanomelissa clementis;* Xyl – *Xylocopa* sp.; Ubee – unidentified bee; Hum – unidentified hummingbird

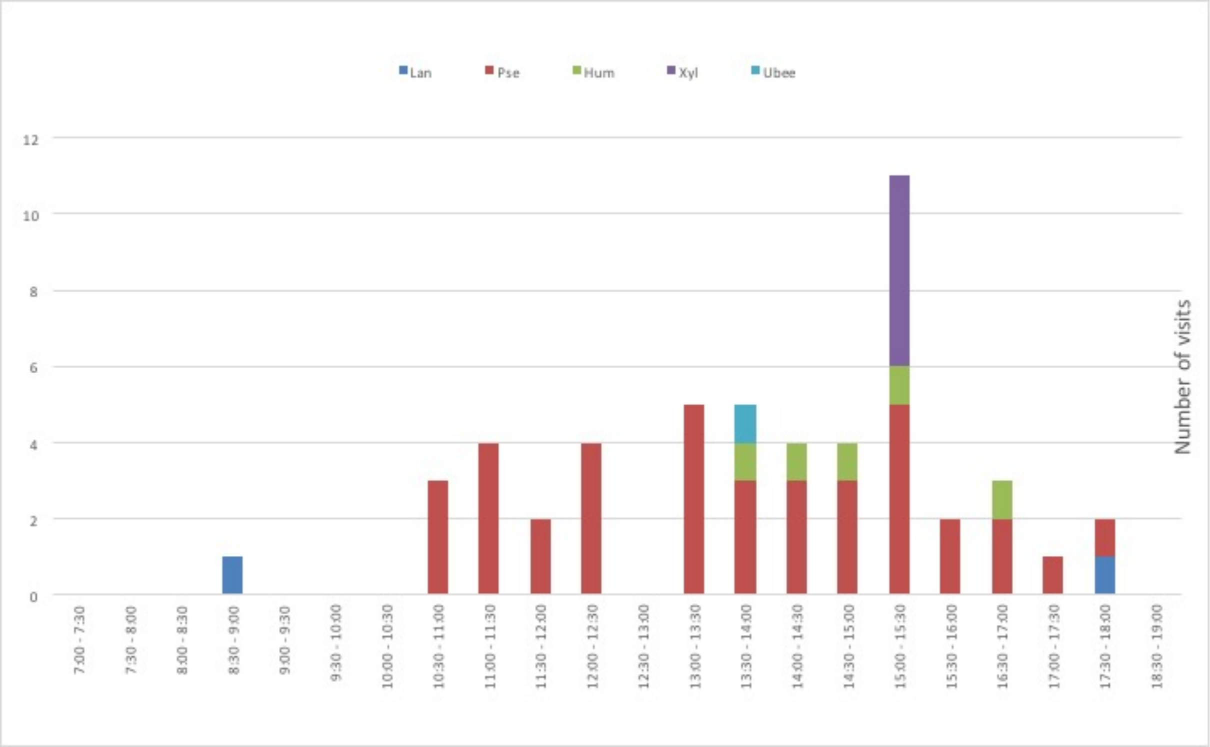

Figure S1. Frequency of visitations in flowers of *Petunia secreta.* Pse: *Pseudagapostemon* sp. (Halictidae); Lan: *Lanthanomelissa clementis* (Apidae); Hum: Unidentified hummingbird (Trochilidae); Xylo: *Xylocopa* sp. (Apidae); Ubee: Unidentified bee (Apidae).
